# The Management of Extrapulmonary Comorbidities and Treatable Traits; Obesity, Physical Inactivity, Anxiety, and Depression, in Adults With Asthma

**DOI:** 10.3389/falgy.2021.735030

**Published:** 2021-09-22

**Authors:** Rebecca F. McLoughlin, Vanessa M. McDonald

**Affiliations:** ^1^National Health and Medical Research Council, Centre of Excellence in Treatable Traits, New Lambton Heights, NSW, Australia; ^2^School of Nursing and Midwifery, Hunter Medical Research Institute, New Lambton Heights, NSW, Australia; ^3^School of Nursing and Midwifery, College of Health, Medicine and Wellbeing, University of Newcastle, Callaghan, NSW, Australia; ^4^Department of Respiratory and Sleep Medicine, John Hunter Hospital, New Lambton Heights, NSW, Australia

**Keywords:** asthma, obesity, physical inactivity, anxiety, depression, treatable traits, extrapulmonary

## Abstract

Asthma is a complex and heterogenous disease characterized by variability in disease expression and severity. Multiple extrapulmonary comorbidities and treatable traits are common in people with asthma, and there is an increasing appreciation of how these may complicate asthma management. This review will discuss the prevalence and impact of extrapulmonary comorbidities/risk factors or “traits,” which have been found to co-exist in asthma (obesity, symptoms of depression and/or anxiety and physical inactivity), the impact these traits have on future outcomes (including exacerbation risk and quality of life) and asthma management, and how we should target treatment in asthma when these extrapulmonary traits are present.

## Introduction

Despite the significant advances in asthma treatment that have been achieved over the past 30 years, improvements in asthma outcomes have stalled, and the burden of asthma from a patient (1), healthcare and economic (2, 3) perspective remains substantial. Around 17% of the asthma population is estimated to have difficult-to-treat asthma (4) which is defined as asthma that is uncontrolled despite medium- or high-dose inhaled corticosteroids (ICS) with a second controller [i.e., long-acting beta2-agonist (LABA) or maintenance oral corticosteroids (OCS)], or that requires high-dose treatment to reduce exacerbations and maintain good symptom control (5). Severe asthma is a subset of difficult-to-treat asthma in which the disease in uncontrolled despite adherence with optimized high-dose ICS-LABA therapy and management of contributory factors, or that worsens when high-dose treatment is decreased (5). Although constituting only 3–8% of the total asthma population, people with severe asthma are disproportionally responsible for much of the burden associated with this disease (4, 6).

It is proposed that the plateauing of improvements in asthma outcome is largely due to the failure of current management approaches to address the complexity and heterogeneity that is evident in asthma (7, 8). Indeed, while individual variability in disease severity, clinical presentation and therapeutic response are increasingly recognized, this is not yet systematically addressed in real-world practice.

To add to this complexity, there are a number of extrapulmonary comorbidities and risk factors, or “traits” that independently affect future asthma outcomes (9–11). This highlights the importance of individualized asthma management strategies that look beyond the pulmonary system. In severe asthma, it has been demonstrated that physical inactivity, symptoms of anxiety and depression, greater systemic inflammation and lower isometric leg strength are independently associated with greater health-related quality of life (HRQoL) impairment (9). While in another analysis, of the 10 predictors of future exacerbation risk identified in severe asthma, eight were extrapulmonary traits; depression, anxiety, obstructive sleep apnea (OSA), vocal cord dysfunction (VCD), upper airway disease, systemic inflammation, underweight, and inhaler device polypharmacy (10).

More recently, Freitas and colleagues conducted a cluster analysis to identify asthma phenotypes based on extrapulmonary traits that are frequently reported in people with moderate to severe asthma, and that are associated with poorer clinical asthma outcomes; physical inactivity and high levels of sedentary time, obesity and psychological disturbances (i.e., symptoms of anxiety and depression) (12). This analysis identified four district clusters: (1) physically active individuals with controlled asthma; (2) physically inactive individuals with uncontrolled asthma who were more sedentary; (3) individuals with uncontrolled asthma who had low levels of physical activity, were obese (body mass index (BMI) ≥30 kg/m^2^) and presented with symptoms of anxiety and depression; and (4) individuals with very uncontrolled asthma, who were physically inactive and highly sedentary, obese, and experienced symptoms of anxiety and depression (12). While participants in clusters 3 and 4 presented with lower health status, greater activity limitation, more asthma symptoms, and greater mental health impairment compared with clusters 1 and 2, cluster 4 was determined to be associated with worse outcomes in terms of exacerbation rates and short-acting bronchodilator use, and had the worst asthma control (12). In addition to demonstrating the different ways in which these extrapulmonary traits co-exist in varying degrees of severity within the asthma population, this study highlights their additive detrimental effects on important asthma outcomes. Furthermore, this reinforces the importance of applying a multidimensional personalized medicine approach to identify and manage these extrapulmonary traits in order to improve asthma management.

This review explores in depth the prevalence and impact of physical inactivity, obesity, and symptoms of anxiety and/or depression in the asthma population, how they may complicate asthma management, and the available evidence on management strategies targeting these extrapulmonary traits in people with asthma.

## The Impact of Extrapulmonary Comorbidities/Traits in Asthma

**Figure 1:**
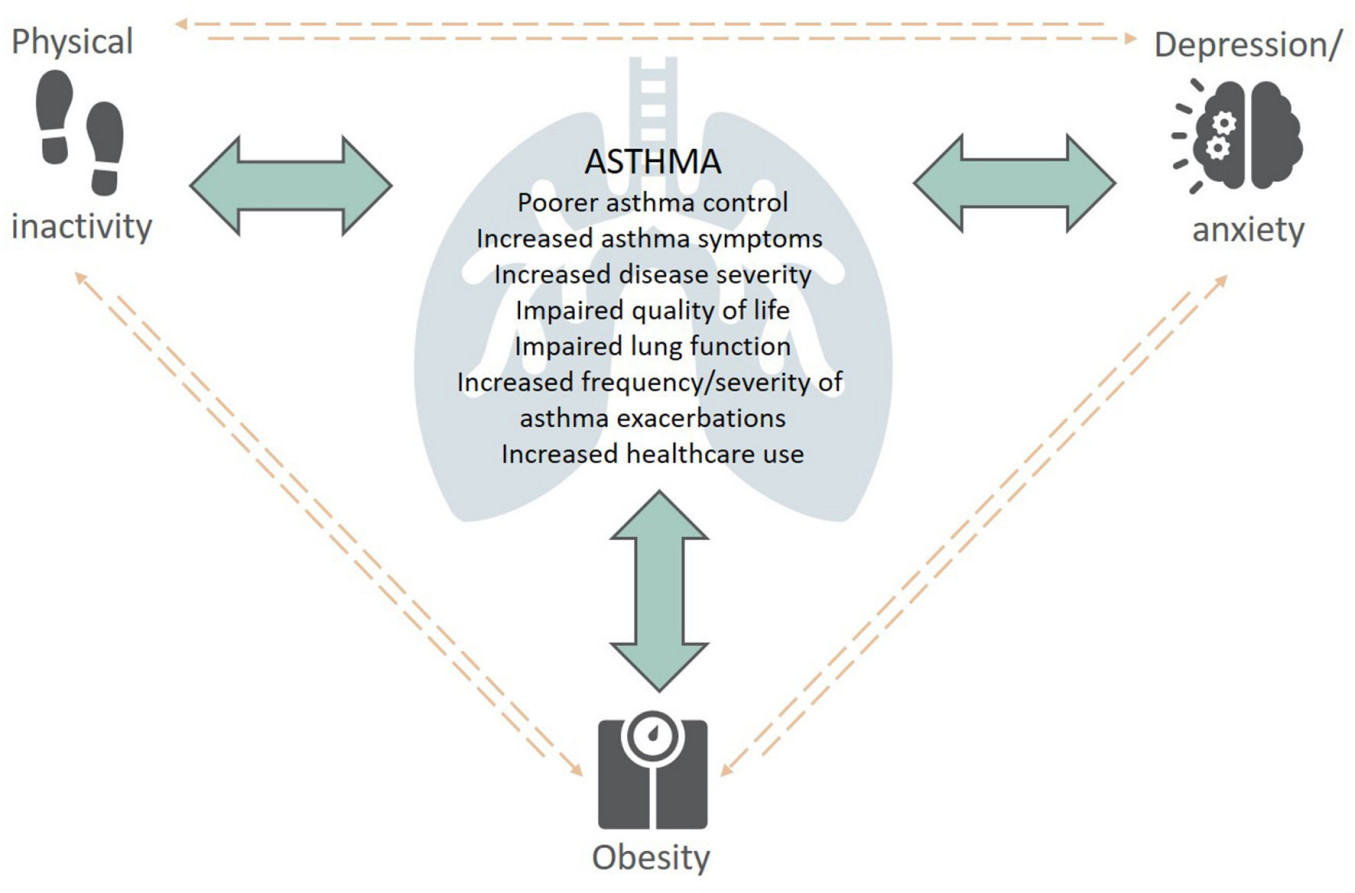
The complex interplay between extrapulmonary traits (physical inactivity, obesity, depression/anxiety) and asthma—Content has been reproduced with permission from the Centre of Excellence in Treatable Traits, originally developed as part of the Centre of Excellence in Treatable Traits (https://treatabletraits.org.au).

There is an increasing appreciation of the impact physical inactivity, obesity and symptoms of anxiety and depression have on the development, severity, clinical presentation, and management of asthma. Given the interrelationship between these extrapulmonary traits (Figure 1), it is not surprising that they often co-exist in people with asthma, with additive deleterious effects (12). For instance, physical inactivity is a well-known risk factor for obesity and is associated with increased levels of anxiety and depression (13), while individuals with obesity are less likely to undertake physical activity (14), sustaining this vicious cycle. There is also evidence of this in individuals with psychological comorbidities, such as anxiety and depression, as they are less physically active which can contribute to weight gain.

### Physical Inactivity in Asthma

Physical inactivity is common in individuals with asthma and is recognized as an important modifiable risk factor for poor clinical outcomes (15). Compared with the general population, individuals with asthma are less likely to engage in physical activity and have been reported to accumulate significantly fewer steps per day (15, 16). In one study, individuals with asthma were reported to spend an average of 60 min less per week undertaking moderate physical activity and an average of 67 min less per week undertaking vigorous physical activity compared to their non-asthma counterparts (17). Low levels of physical activity are particularly evident in the severe asthma population. In a study by Cordova-Rivera and colleagues (18), patients with severe asthma were reported to undertake significantly less moderate and higher intensity activity [median (IQR) 21.9 (12.9–36.0) vs. 41.7 (29.5–65.2) min per day (*P* < 0.0001)], and accumulated 2,232 fewer steps per day than their age and sex-matched asthma counterparts.

Observational data suggest that individuals with asthma avoid physical activity due to a fear of provoking exercise-induced bronchoconstriction (EIB) (19, 20), which is an acute narrowing of the airways that occurs with exercise (21). EIB is characterized by asthma symptoms including, wheeze, shortness of breath and cough, and is estimated to occur in ~90% of individuals with asthma (19). Although exercise is a known trigger for asthma, people with asthma can safely achieve the physical activity recommendations for the general population (20–60 min of physical activity 3–5 days per week) by actioning strategies to prevent EIB. These strategies include pre-treatment with a bronchodilator and undertaking adequate warm-up and cool-down exercises (5, 22, 23).

There are many detrimental consequences associated with physical inactivity in individuals with asthma. In addition to the adverse health consequences experienced by the general population, physical inactivity in asthma is associated with increased disease severity, poorer asthma control and respiratory functioning, increased exacerbation risk, increased healthcare use, impaired quality of life, and decreased physical and mental health (13). Furthermore, physical inactivity and the adoption of a sedentary lifestyle are associated with deconditioning (24). This can result in a vicious cycle of declining physical activity, which leads to decreased physical fitness and lowers an individual’s threshold for EIB (24). Physical inactivity is also a well-known risk factor for obesity, which has also been shown to negatively impact asthma outcomes (25).

Conversely, higher levels of physical activity in asthma have been associated with better asthma control, lung function and exercise capacity and decreased markers of systemic inflammation (15, 18, 26). In one study, compared to individuals with asthma who were inactive (defined as an energy expenditure <1.5 kcal/kg body weight (BW) per day) or moderately active (1.5 kcal/kg BW per day), individuals with asthma who were active (>3.0 kcal/kg BW per day), were reported to have greater overall health, less mental health impairments, fewer activity limitations and better perceived mental health (27).

### Obesity in Asthma

In 2017–18, 42% of Australian adults with asthma had comorbid obesity, with obesity rates higher in people with asthma than in the general population (~30%) (28). Over recent decades, the increasing prevalence of obesity and asthma have paralleled one another, with evidence suggesting a bi-directional association between the two chronic conditions. That is, asthma may increase the risk of obesity (29–31) and vice versa (32). In one longitudinal study with 10-years follow-up (*n* = 2,171) the risk of developing obesity during childhood and adolescence was reported to be 51% higher in children with asthma compared to those without asthma (30). Similarly, a pooled analysis of 16 European cohorts (*n* = 21,130) found that asthma increased the relative risk of obesity by 1.7-fold (33). As mentioned earlier, this is not surprising given the association between asthma and reduced physical activity, which is a risk factor for obesity. Obesity, on the other hand, is also recognized as an independent risk factor for asthma (32, 34). In a meta-analysis conducted in >300,000 adults, obesity was found to be associated with both increased asthma prevalence and incidence (particularly in females), with the odds of incident asthma increased by 90% in adults with obesity (32).

While the underlying mechanisms driving the association between asthma and obesity are yet to be completely elucidated, it has been proposed that multiple mechanisms and overlapping modulators are involved (35). Pathophysiological factors that may be implicated include metabolic and microbiome dysfunction (36), obesity-related local and systemic inflammation (36), genetic predisposition (37), increased mechanical load/stress on the lungs (38) and obesity-related hormonal changes such as an increase in leptin; an adipocyte-derived hormone which has been found to increase airway inflammation (37). Lifestyle factors including poor diet quality, physical inactivity and over-nutrition are also suggested to contribute (39).

Obesity is increasingly recognized as a disease modifier in asthma, which subsequently complicates asthma management. Compared with non-obese people with asthma, individuals with comorbid obesity and asthma appear to have more difficult to control disease (36) with worsened asthma symptoms (such as dyspnoea and wheezing) (40), fewer symptom-free days (41, 42), lower exercise capacity (43), reduced lung function (44), and lung volumes (45), increased frequency and severity of asthma exacerbations (36), increased risk of asthma-related hospitalizations (46), and poorer asthma-related quality of life (QoL) (47). In fact, BMI has been found to be an independent predictor of poor QoL (48), with QoL more than five times worse in people with asthma who are obese compared to those who are non-obese (49). Individuals with asthma and comorbid obesity are also at an increased risk of multimorbidities such as anxiety, depression and obstructive sleep apnea, which may also worsen asthma control (50). Whether obesity increases asthma severity however remains unclear, with conflicting evidence reported in the literature (42, 49, 51, 52).

Individuals with comorbid asthma and obesity have also been found to use more asthma medications compared to those without obesity (53). In a recent systematic review and meta-analysis by Thompson and colleagues, obesity was found to be associated with increased odds of any asthma medication use, which included inhaled bronchodilators [short-acting (SABA) and long-acting (LABA, anticholinergic)], inhaled controller medications [inhaled corticosteroids (ICS, as well as combined ICS and LABA)], and oral preventer medications [maintenance oral corticosteroid (OCS), Leukotriene receptor antagonists (LTRA)] (44). Daily ICS doses were also significantly higher in obese individuals with asthma compared with their healthy-weight (BMI <25 kg/m^2^) counterparts (44).

Of particular interest when considering the implications of managing individuals with comorbid obesity is the growing body of evidence that individuals with obesity have a reduced response to asthma medications (36, 54–56). While the mechanisms responsible remain largely unknown, one explanation is that it is related to the increased production of obesity-related pro-inflammatory cytokines, which are suggested to reduce glucocorticoid induction of mitogen-activated kinase phosphatase 1; a signaling protein that plays an integral role in steroid responses (57). Despite this, asthma management guidelines do not currently differentiate pharmacotherapy medication choices or dosages for individuals with asthma and comorbid obesity. This is a major clinical problem that further adds to the complexity of asthma management in individuals with comorbid obesity.

### Anxiety and Depression in Asthma

Psychological comorbidities are highly prevalent in the asthma population, with anxiety and depression reported to be up to six times more common in people with asthma than those without (58). Prevalence rates of 11–37% for anxiety and 11–18% for depression have been reported in people with asthma (59), with these psychological comorbidities often found to co-exist (60). While the association between these psychological comorbidities and asthma severity remains controversial (61), prevalence rates of anxiety and depression have been reported to be even higher among the severe asthma population (38 and 25%, respectively) (10). In one study, anxiety was reported to be 1.4 times more common and depression 3.3 times more common in people with severe asthma compared to those with non-severe disease (10).

Despite the high prevalence of anxiety and depression in people with asthma, these psychological comorbidities are often underdiagnosed and under-treated in clinical practice. In a systematic review of asthma multidimensional assessment, it was identified that psychological health is only assessed in two-thirds of severe asthma patients (62). Similarly, in a study examining the relationship between anxiety and asthma management (*n* = 201), although 51.5% of participants were determined to have clinically significant levels of anxiety (Beck Anxiety Inventory (BAI) score ≥ 16), only 21% of these individuals had been formally diagnosed with anxiety and were receiving treatment in clinical practice (63). Anxiety and asthma share a number of similar symptoms including, chest tightness, shortness of breath, rapid breathing/hyperventilation and feelings of lightheadedness (64). This symptom overlap makes it difficult to differentiate between the two conditions and has been suggested to contribute to the overall underdiagnoses and under-treatment of anxiety disorders in individuals with asthma (65). Other possible reasons for the underdiagnoses and under-treatment of both anxiety and depression in asthma include sub-optimal patient-clinician communication, insufficient consultation times for clinicians to make an appropriate diagnosis, limited access to mental health services, and patient-related barriers relating to the stigma of mental illnesses (63).

The nature of the association and the direction of causality between asthma and comorbid anxiety/depression is complex. While some studies have suggested that anxiety and depression are associated with a higher risk of developing asthma (60, 66–68), others have proposed that asthma, particularly if it is poorly controlled, may be the cause of subsequent psychological comorbidities (69). Irrespective of the direction of causality however, the presence of these psychological comorbidities in asthma are associated with poorer asthma control (68), increased asthma symptoms (63, 70), greater frequency of exacerbations (68), higher corticosteroid dosages (67, 71) and greater rates of non-adherence to asthma treatment (72). It is therefore not surprising that compared to asthma alone, comorbid anxiety and depression in asthma is associated with greater healthcare use including, unscheduled general practitioner visits, emergency department presentations and hospital admissions (63, 73, 74), with one study reporting that comorbid depression and anxiety in asthma was associated with 51% higher healthcare costs (14, 70, 75). While evidence regarding the association between anxiety/depression and lung function in asthma is conflicting (63, 73, 74), the adverse effect of these psychological comorbidities on QoL is well-documented (14, 70, 75). A recent systematic review by Stanescu and colleagues demonstrated that anxiety and depression are consistently reported to have a substantial impact on both general and asthma-related QoL (14). In one study, asthma-related QoL was found to be 1.4 times lower in individuals with comorbid asthma and depression compared to those with asthma alone (76).

The underlying mechanisms responsible for the associations between depression and anxiety and poor clinical outcomes in individuals with asthma are complex and are proposed to involve inflammatory processes. Individuals with depression are reported to have increased levels of pro-inflammatory cytokines including, interleukins (IL)-1, IL-4, and IL-6 and TNF-alpha, which are known to play important roles in the pathogenesis of asthma (77). Anxiety, on the other hand, has been suggested to affect asthma control by increasing parasympathetic activity and the secretion of pro-inflammatory cytokines (78).

There are several ways in which comorbid anxiety and depression may complicate asthma management. In individuals with asthma, it has been demonstrated that anxiety is associated with an increased subjective perception of respiratory symptoms such as shortness of breath, in the absence of any change in airway obstruction measured objectively (79, 80). Furthermore, due to the symptom overlap between anxiety and asthma, symptoms of anxiety may be misinterpreted by patients and clinicians as asthma symptoms (65). This may lead to an overuse of asthma medications by the individual and potentially an incorrect physician assessment of asthma control resulting in a higher medication prescription (79). Indeed, higher levels of anxiety in individuals with asthma have been positively correlated with high doses of inhaled glucocorticoids (63).

Psychological comorbidities can also impair an individual’s confidence in their ability to make appropriate self-management decisions. Indeed, anxiety (81) and depression (76, 82–85) have been identified as important risk factors for poor treatment adherence; one of the main contributors to suboptimal asthma management and subsequently poor clinical outcomes (86). In one study, high levels of depressive symptoms were associated with an 11-fold increase in odds of poor adherence to inhaled corticosteroid therapy (58). Furthermore, depression is known to have a significant impact on an individual’s motivation to engage in health-promoting behaviors. Consequently, individuals with asthma and comorbid depression are also more likely to be non-adherent to behavior change advice such as smoking cessation, allergen avoidance and increasing physical activity, increasing their likelihood of exacerbations and poor asthma control (87).

Depression has also been demonstrated to decrease bronchodilator response (BDR) in individuals with asthma. In a nationwide study of 20,272 adults from the 2007–2012 National Health and Nutrition Examination Survey, major depression (Patient Health Questionnaire (PHQ-9) score ≥15) was reported to be associated with a 4.2% reduction in bronchodilator response (BDR) in asthma (88). This finding is supported by a recent study in which depressive symptoms (Hospital and Anxiety score ≥8) were associated with a 6.52% reduction in BDR (89). In this study, depressive symptoms were also associated with a 13.38% elevation in sputum neutrophils (89). Given that neutrophilic airway inflammation has been shown to contribute to corticosteroid resistance, this may further complicate the management of individuals with asthma and comorbid depression.

## Managing Asthma With Extrapulmonary Comorbidities/Traits

The significant impact of physical inactivity, obesity, and psychological comorbidities in individuals with asthma, and the ways in which they complicate asthma management, highlights the need to address these extrapulmonary comorbidities using evidence-based interventions.

### Management of Physical Inactivity in Asthma

In a systematic review, Cordova-Rivera and colleagues concluded that higher levels of physical activity are associated with better asthma control, lung function and health status and less exacerbations and health care use (15). Furthermore, there is increasing evidence in the literature regarding the benefits of exercise interventions (particularly aerobic exercise) in individuals with asthma. Indeed, current research suggests that engaging in moderate-intensity aerobic exercise for at least 30 min, 2–5 times per week, improves both physiological and psychological outcomes in this population (90, 91). However, few studies to date have reported on the effectiveness of interventions in increasing physical activity in individuals with asthma (90). This is an important gap in the literature that needs to be addressed to determine the most effective strategy for managing physical inactivity in asthma.

#### Exercise Interventions

Evidence on the impact of exercise interventions on physical activity measures, whether this is daily steps or time spent undertaking physical activity, is scarce. A RCT, conducted in grade II obese adults (BMI ≥35 and <40 kg/m^2^) with moderate to severe asthma (*n* = 55), studied the effect of exercise training (supervised aerobic and resistance exercise) combined with a weight loss program (nutrition and psychological therapies) on physical activity and comorbidities, compared with a weight loss program alone. After 3 months, the exercise training and weight loss program resulted in a greater improvement in physical activity (daily steps, time spent in light-intensity physical activity and MVPA), the number of asthma symptom-free days, sleep efficiency and symptoms of depression (92). More recently, Evaristo and colleagues investigated the effects of aerobic training compared with breathing exercises (Pranayama Yoga breathing technique) for 3 months on asthma control in adults with moderate to severe asthma (*n* = 54), with physical activity (daily steps quantified using an accelerometer) measured as a secondary outcome. Interestingly, no significant between-group change in physical activity was reported, with participants in both groups increasing their physical activity by ~2,000 steps. Furthermore, after 3 months, participants in both groups were achieving the 10,000 steps per day recommendation for the general population. To our knowledge, no studies to date have investigated the long-term effect of structured exercise training on physical activity in asthma. This is an important knowledge gap as changing physical activity behavior requires a long-lasting approach (93).

#### Behavioral Interventions

Conscious behavior change is required to increase engagement in physical activity (90). Behavior change can be achieved using techniques such as setting personalized goals, developing “ behavior contracts,” identifying motivators to change, increasing an individual’s knowledge of why the behavior change is important and beneficial, and using self-monitoring combined with feedback (94). To date, few studies have examined the use of behavior change interventions to increase physical activity in individuals with asthma (90). In one study, adults with mild to moderate asthma (*n* = 258) were randomized to receive either asthma education and physical activity goal setting (control) or the same intervention combined with self-affirmation and positive-effect components to promote self-efficacy (multi-component intervention). While a greater increase in physical activity (energy expenditure) was observed with the multi-component intervention [415 (95% CI, 76–754 kcal/week) vs. 398 (95% CI, 145–652 kcal/week)], there was no significant difference in change between groups after 12 months (*P* = 0.94) (95). However, this study used self-reported methods to assess physical activity, which may have introduced measurement bias. In another study that used wearable technology to measure physical activity, a 12-week step count-based intervention (using a pedometer for physical activity monitoring and goal setting) was reported to be effective in increasing physical activity (daily steps) in adults with moderate to severe asthma, with a significant between-group difference reported [MD [95% CI], 2,488 steps [803 to 4,172]]. No improvements in asthma control were observed however, which may suggest that a greater increase in daily steps is required to improve asthma control (96).

More recently, Freitas and colleagues examined the use of an 8-week comprehensive behavior change intervention to increase physical activity in individuals with moderate to severe asthma who were physically inactive (26). In this study, motivational interviewing was used to determine the participants “readiness to change,” followed by the application of a range of behavior change techniques to increase physical activity and reduce sedentary time, and supported by a workbook and an activity monitor (26). Compared to usual care, the comprehensive behavior change intervention was reported to significantly improve physical activity (daily steps, sedentary time, time spent in moderate to vigorous activity), asthma control, anxiety symptoms and sleep quality in adults with moderate to severe asthma (26). Furthermore, there was an inverse association between change in time spent undertaking moderate-intensity physical activity and change in asthma control (26). These findings highlight the potential for comprehensive behavior change interventions to be used in the asthma population to improve not only physical activity, but also clinical control.

#### Pharmacotherapy

Biologics, primarily monoclonal antibodies (i.e., omalizumab, mepolizumab, benralizumab, and dupilumab), are a relatively new add-on treatment option for severe asthma which target specific inflammatory molecules and pathways important in the pathogenesis of asthma (5, 97). These monoclonal antibodies have been shown to improve asthma symptom control, quality of life and lung function, and reduce asthma exacerbation frequency, OCS use and healthcare utilization (97). The effect of pharmacological therapies such as biologics on physical activity levels in asthma, however, is largely unknown. To our knowledge, Carpagnano and colleagues were the first to explore the effect of biologics on physical activity levels in severe asthma compared to traditional therapies (98). In this pilot study, published in 2020, fifty patients with severe asthma who were treated with either high-dose ICS with a LABA/LAMA and a biologic [omalizumab (*n* = 15) or mepolizumab (*n* = 15)], or traditional therapy (ICS/LABA/LAMA/OCS, *n* = 20) were followed over 6 months (98). While improvements in physical activity levels [average steps per day (SPD) and total daily energy expenditure (Total EE)] were observed both groups (biologic therapy and traditional therapy), a significantly greater increase in physical activity was reported in patients who received a biologic add-on therapy (98). No significant differences in physical activity levels were reported in patients who were received omalizumab compared to mepolizumab, with both biologics found to have comparable positive effects. While this pilot study provides promising evidence that a single add-on therapy can significantly improve physical activity levels in patients with severe asthma, further research is needed to explore these findings.

### Management of Obesity in Asthma

The adverse consequences of comorbid obesity in individuals with asthma, combined with the increasing prevalence of obesity in asthma highlight the need for appropriate management strategies in this population. While it has been demonstrated that significant improvements in asthma outcomes can be achieved with weight loss of 5–10% (99–102), there is currently a paucity of evidence from high-quality studies to guide clinicians, with the most optimal method of achieving weight loss in this population largely unknown. Therefore, the obesity management guidelines used for the general population are currently followed for individuals with asthma and comorbid obesity (103).

**Figure 2:**
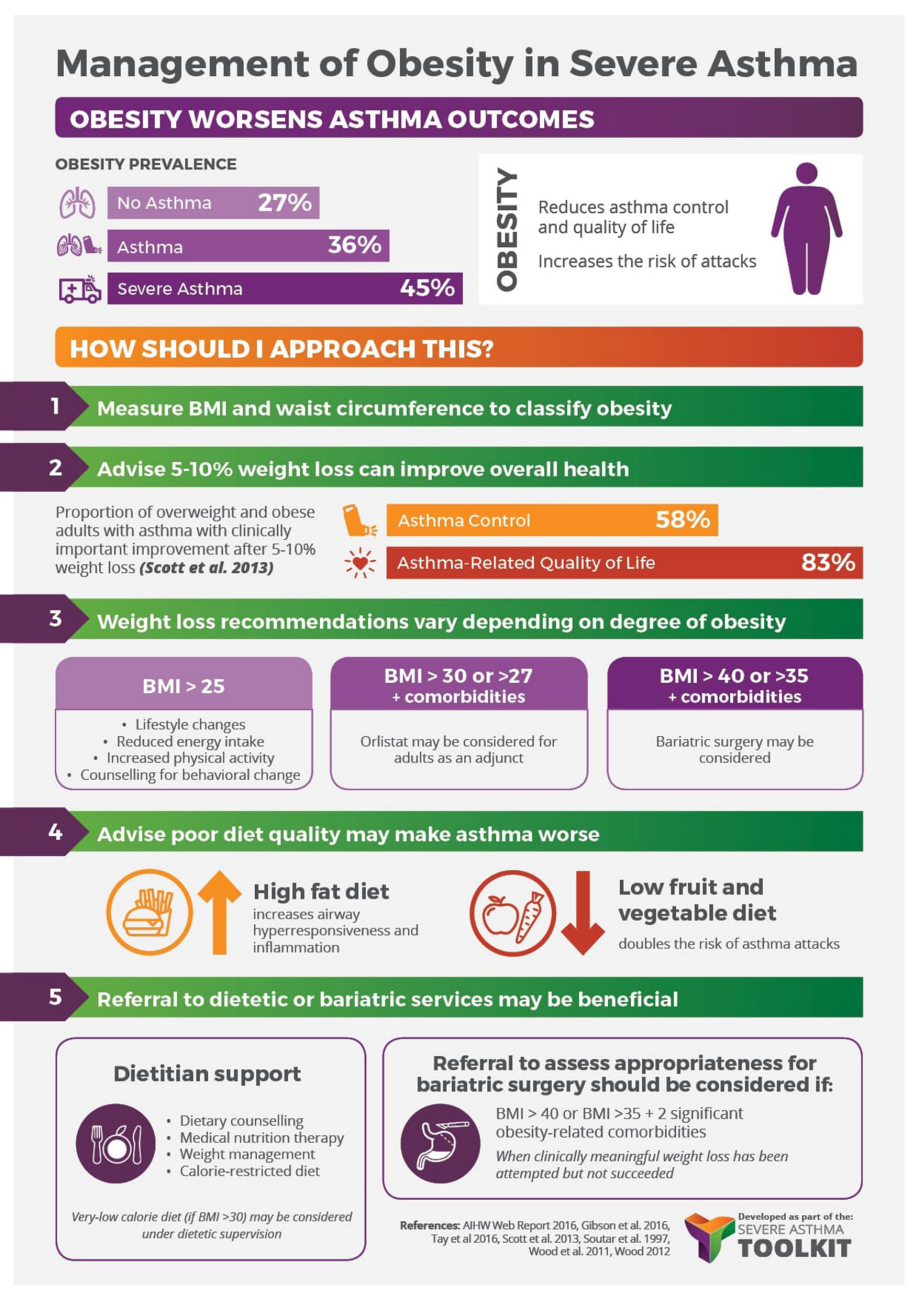
Management of obesity in severe asthma infographic—Content has been reproduced with permission from the Centre of Excellence in Severe Asthma, originally developed as part of the Centre of Research Excellence in Severe Asthma (https://toolkit.severeasthma.org.au).

A variety of weight-loss strategies are used in the general population, guided by the individual’s degree of obesity and using an additive approach (103). These include lifestyle interventions (BMI ≥ 25 kg/m^2^, i.e., diet/calorie restriction, exercise, combined diet and exercise, behavior change counseling), pharmacotherapy (BMI ≥30 or ≥27 kg/m^2^ if comorbidities are present. i.e., orlistat) and bariatric surgery (BMI >40 or >35 kg/m^2^ if comorbidities are present). The following summarizes the current evidence on the use of these weight-loss strategies in the asthma population (Figure 2).

#### Lifestyle Medicine

Lifestyle interventions for weight-loss focus on increasing physical activity and reducing energy intake through diet, exercise, and behavior change, and are recommended for all individuals with overweight or obesity (BMI≥25 kg/m^2)^. A variety of weight-loss interventions have been trialed in individuals with asthma including dietary/calorie restriction, exercise interventions and combined dietary restriction and exercise/behavioral therapies. Several reviews summarizing the effectiveness of these interventions in the asthma population have been published (39, 102, 104, 105). In one review, weight loss in obese individuals with asthma was found to be associated with a 48–100% improvement in asthma symptoms, as well as an improvement in asthma medication use, asthma severity, exercise tolerance, dyspnoea, exacerbations, and asthma-related hospitalizations (105). Similarly, a more recent systematic review by Okoniewski and colleagues, which involved 4 RCTs in children (*n* = 246) and 6 RCTs in adults (*n* = 02), concluded that irrespective of the type of intervention used (i.e., dietary restriction, exercise or behavioral therapy), weight-loss generally resulted in improved asthma-related quality of life and asthma control (104).

However, due to the high heterogeneity between studies, it is difficult to determine the most optimal lifestyle weight-loss strategy for this population. This is further complicated by the conflicting evidence in the literature. For example, in a study conducted by Scott and colleagues comparing three weight-loss interventions (dietary restriction alone, exercise alone or combined exercise and dietary restriction) in overweight/obese adults with asthma, greater weight loss was reported in the dietary and combined diet and exercise groups (8.5 and 8.3%, respectively), compared to exercise alone (1.8%) (99). Conversely, in a trial by Freitas and colleagues, greater weight loss was reported in the combined diet and exercise (weight-loss program) group, compared with the group who received a diet and sham activity intervention (106). Nonetheless, these findings demonstrate that lifestyle interventions used in the general population are also effective in achieving weight loss in the asthma population, which improves asthma outcomes including, asthma-related quality of life and asthma control.

#### Pharmacotherapy

Weight loss pharmacotherapy is recommended in individuals with a BMI ≥30 kg/m^2^ (or ≥27 kg/m^2^ if comorbidities are present, i.e., diabetes or hypertension) as an add-on treatment if weight loss has not been achieved after 6 months of using lifestyle interventions (103). To our knowledge, only one weight loss pharmacotherapy study in individuals with asthma has been conducted. This study examined the effect of weight loss induced by a medical weight loss program [dietary restriction combined with two weight loss medications (Sibutramine and Orlistat) for 6-months, on obese individuals (BMI ≥30 kg/m^2^)] with severe asthma (100). Participants achieved an average weight loss of 7.88 kg (7.5% weight loss), which was associated with significant improvements in lung function, asthma control and asthma symptoms, and a decrease in reliever medication use (100). However, while orlistat, a lipase inhibitor that reduces fat absorption, is approved by the US Food and Drug Administration (FDA), the appetite suppressant Sibutramine is no longer an FDA-approved drug and has been discontinued in many countries.

#### Bariatric Surgery

Improvements in several asthma outcomes including, airway hyperresponsiveness (AHR), asthma severity, lung function, asthma control and asthma-related quality of life have been reported following weight loss associated with bariatric surgery (35, 107–110). Bariatric surgery has also been shown to reduce asthma-related hospitalizations and asthma medication use (111–113). In one study involving adult bariatric patients prescribed at least one asthma medication (*n* = 751, mean BMI 49 ± 8.2 kg/m^2^), bariatric surgery was reported to significantly reduce asthma medication use 30 days post-surgery, with this reduction sustained for up to 3 years (113). Similar reductions in medication use regardless of bariatric surgery procedure type (i.e., Roux-en-Y gastric bypass, sleeve gastrectomy, adjustable gastric banding, and duodenal switch) (113). Reddy and colleagues also found a decrease in asthma medication use in obese asthma patients (*n* = 257, mean BMI 49 ± 10 kg/m^2^) 1 year post-bariatric surgery; ~46% of patients who initially needed OCS for symptom control no longer requiring them, while the percentage of patients using ICS decreased from 49.8 to 29.6% (112). Furthermore, although this study demonstrated that bariatric surgery, in general, was effective in achieving weight loss, with a mean weight loss of 56% at 1-year follow-up, laparoscopic adjustable gastric banding (LAGB) was found to be the least effective and was less likely to reduce asthma medication use (112).

While there is evidence that individuals with asthma and comorbid obesity may benefit from bariatric surgery-induced weight loss, there remains a paucity of evidence to support the use of bariatric surgery in these individuals in the absence of other comorbidities. This is consistent with the current obesity management guidelines for the general population. Further high-quality studies are needed to inform asthma management guidelines in relation to the use of bariatric surgery in the asthma population.

### Management of Anxiety and Depression in Asthma

In the general population, a stepped care approach for the management of anxiety and depression is recommended, whereby individuals are started on low-intensity treatments and are progressively moved up to more intensive treatments until symptom relief is achieved (114). Standard treatment approaches used in the general population include lifestyle/behavior interventions (i.e., diet, exercise, weight loss, psychoeducation, meditation, management of risk factors), psychological therapy [i.e., cognitive behavioral therapy (CBT) and behavior therapy], pharmacotherapy [i.e., antidepressants such as serotonin and norepinephrine reuptake inhibitors (SNRIs), selective serotonin reuptake inhibitors (SSRIs), tricyclic antidepressants, and anxiolytics such as benzodiazepines], and in some cases requiring more intensive treatment, electroconvulsive therapy (ECT) (114).

Individuals with asthma and comorbid anxiety and depression are currently treated using the same stepped care approach used in the general population. Studies on the management of anxiety and depression in people with asthma using these treatment approaches however, are limited (115), particularly in those with severe asthma.

#### Lifestyle Medicine

This expanding field of medicine involves the use of evidence-based therapeutic interventions such as dietary modification, physical activity/exercise, stress management (i.e., medication, breathing training, relaxation), restorative sleep, and the reduction or avoidance of recreational substances, which address key risk factors, in order to prevent and treat chronic diseases including psychological comorbidity (116). The main lifestyle medicine interventions that have been studied to date in individuals with asthma for the management of anxiety and depression include; physical activity/exercise interventions (92, 96, 115, 117–119), breathing and relaxation training (120–124), imagery (125, 126), and spiritual healing (127).

Engaging in regular physical activity or exercise is known to promote psychological well-being in the general population. To date, the majority of physical activity/exercise intervention studies conducted in individuals with asthma support the beneficial role of increased physical activity in reducing symptoms of anxiety and depression, increasing symptom-free days and improving health-related quality of life (92, 117–119). Breathing and relaxation training interventions have shown promise in reducing anxiety and depression scores in individuals with asthma (120–124), however there is limited evidence regarding the benefits of imagery instruction (125, 126) and spiritual healing (127). While many of the individuals included in these studies had symptoms of anxiety and/or depression, there are a lack of studies examining the benefits of these lifestyle medicine interventions in individuals with asthma and underlying comorbid anxiety and/or depressive disorders.

#### Psychological Interventions

Psychological interventions have shown the most promise in the management of psychological comorbidities in individuals with asthma. Several reviews have demonstrated there is increasing evidence that psychological interventions, particularly cognitive behavioral therapy (CBT) and relaxation therapy, are beneficial in improving not only psychological outcomes (i.e., anxiety, depression, fear, panic) individuals with asthma, but also a number of asthma-related outcomes (i.e., exacerbations, symptoms and asthma-related quality of life) (115, 128, 129). However, high heterogeneity has been identified between studies conducted to date.

A recent systematic review of 12 studies focusing specifically on CBT for the management of anxiety in asthma concluded that while there is preliminary evidence to support the use of CBT for anxiety management in adults with asthma, more high-quality randomized controlled trials of longer duration are needed (130). Similarly, while evidence for the use of CBT in children with asthma for the management of anxiety is promising, this has been under-studied and warrants further attention. An important finding of this review, however was that anxiety-focused CBT interventions were found to be more beneficial than generic CBT interventions focusing on illness perception (130). Further high-quality is needed to inform asthma management guidelines on the management of individuals with comorbid psychological comorbidities.

#### Complementary and Alternative Medicine (CAM)

In the scoping review conducted by Cooley and colleagues, one study examining the effect of CAM treatments (acupuncture alone, craniosacral therapy alone, or combined acupuncture and craniosacral therapy) on symptoms of anxiety and depression in asthma was identified. Both acupuncture alone and craniosacral therapy alone was found to significantly improve asthma-related quality of life, however there was no significant effect on symptoms of anxiety and depression compared to control (131). Larger randomized controlled trials are needed to determine the benefits of CAM in the management of anxiety and depression in individuals with asthma.

#### Pharmacotherapy

As highlighted by Cooley and colleagues (115), and more recently in a systematic review and meta-analysis by Tran and colleagues (59), the pharmacological management of anxiety and depression in individuals with asthma remains under-investigated (59, 115). Tran and colleagues identified six randomized controlled trials investigating the effectiveness and safety of pharmacological interventions in the treatment of psychological distress (primarily major depressive disorder) in individuals with asthma (132–136). Of these, four evaluated the effectiveness of a SSRI [*n* = 2 used Citalopram (134) (Heaney, unpublished, 2018) and *n* = 2 used Escitalopram (132, 133)] compared to placebo, one studied the use of an anti-epileptic medication (Levetiracetam) (135), and one used an atypical antidepressant (Tianeptine) (136). Meta-analysis of pooled data from the four SSRI studies (132–134) showed no significant effect of SSRIs on depressive symptoms compared to control. Similarly, compared to control, no difference in depressive symptoms were reported in individuals treated with Levetiracetam (135). From these reviews (59, 115), it is evident that there is currently a paucity of evidence regarding the role of pharmacotherapy in the management of depression in individuals with asthma.

Furthermore, anxiolytic medications (i.e., benzodiazepines) commonly used to treat anxiety have been reported to be associated with adverse respiratory effects such as respiratory depression (137, 138), limiting their clinical usefulness in patients with asthma. Indeed, it is due to this adverse side effect that anxiolytic medications should be avoided during asthma exacerbations (5). Furthermore, in one study, current Benzodiazepine use was found to increase the likelihood of asthma exacerbations by 49%, and the likelihood of mortality following exacerbation by 32% (138), however more studies are needed to explore this association.

## Summary and Future Directions

There is an increasing appreciation of the impact extrapulmonary traits have on asthma outcomes and the asthma management challenges that may arise when these traits are present. Management strategies targeting physical inactivity, obesity, and depression/anxiety, however, have been under-investigated in the asthma population, and more so in people with severe asthma. Nonetheless, there is promising evidence that managing these traits improves important clinical outcomes in asthma, reinforcing the importance of individualized asthma management strategies that look beyond the pulmonary system. High-quality studies are urgently needed to determine the most optimal strategies to manage these extrapulmonary traits in individuals with asthma. Given the clustering of these extrapulmonary traits and the multiplicative deleterious impacts when these traits co-exist, bundled multi-component interventions targeting physical inactivity, obesity and psychological health require investigations, especially in those with severe disease where the impacts are greatest.

## Permission to Reuse and Copyright

Management of obesity in severe asthma infographic — Content has been reproduced with permission from the Center of Excellence in Severe Asthma, originally developed as part of the Center of Research Excellence in Severe Asthma (https://toolkit.severeasthma.org.au).

## Author Contributions

Both authors (RM, VM) contributed to the conceptualization and writing of the manuscript and have reviewed and approved the final submitted version.

## Conflict of Interest

The authors declare that the research was conducted in the absence of any commercial or financial relationships that could be construed as a potential conflict of interest. The handling Editor declared a past co-authorship with one of the authors (VM).

## Publisher’s Note

All claims expressed in this article are solely those of the authors and do not necessarily represent those of their affiliated organizations, or those of the publisher, the editors and the reviewers. Any product that may be evaluated in this article, or claim that may be made by its manufacturer, is not guaranteed or endorsed by the publisher.
